# Transcriptome Analysis Reveals the Profile of Long Non-coding RNAs During Chicken Muscle Development

**DOI:** 10.3389/fphys.2021.660370

**Published:** 2021-05-10

**Authors:** Jie Liu, Yan Zhou, Xin Hu, Jingchao Yang, Qiuxia Lei, Wei Liu, Haixia Han, Fuwei Li, Dingguo Cao

**Affiliations:** ^1^Poultry Institute, Shandong Academy of Agricultural Sciences, Jinan, China; ^2^Poultry Breeding Engineering Technology Center of Shandong Province, Jinan, China; ^3^Molecular and Cellular Biology, Gembloux Agro-Bio Tech, University of Liège, Gembloux, Belgium; ^4^Shandong Animal Husbandry General Station, Jinan, China

**Keywords:** chicken, breast muscle, development, lncRNA, co-expression

## Abstract

The developmental complexity of muscle arises from elaborate gene regulation. Long non-coding RNAs (lncRNAs) play critical roles in muscle development through the regulation of transcription and post-transcriptional gene expression. In chickens, previous studies have focused on the lncRNA profile during the embryonic periods, but there are no studies that explore the profile from the embryonic to post-hatching period. Here, we reconstructed 14,793 lncRNA transcripts and identified 2,858 differentially expressed lncRNA transcripts and 4,282 mRNAs from 12-day embryos (E12), 17-day embryos (E17), 1-day post-hatch chicks (D1), 14-day post-hatch chicks (D14), 56-day post-hatch chicks (D56), and 98-day post-hatch chicks (D98), based on our published RNA-seq datasets. We performed co-expression analysis for the differentially expressed lncRNAs and mRNAs, using STEM, and identified two profiles with opposite expression trends: profile 4 with a downregulated pattern and profile 21 with an upregulated pattern. The *cis-* and *trans-*regulatory interactions between the lncRNAs and mRNAs were predicted within each profile. Functional analysis of the lncRNA targets showed that lncRNAs in profile 4 contributed to the cell proliferation process, while lncRNAs in profile 21 were mainly involved in metabolism. Our work highlights the lncRNA profiles involved in the development of chicken breast muscle and provides a foundation for further experiments on the role of lncRNAs in the regulation of muscle development.

## Background

The developmental complexity of muscle arises from elaborate gene regulation. A precise map of transcripts, together with their expression dynamics in the developmental stages of muscle, can provide molecular insights into muscle growth and development. Data from high-throughput sequencing studies have revealed that protein coding sequences constitute a small proportion of the whole genome and the majority of sequences are transcribed as non-protein coding RNAs (ncRNAs) (Muers, 2011; Djebali et al., 2012). The ncRNAs that are shorter than 200 nucleotides are usually described as small/short ncRNA and include microRNAs, PIWI-interacting RNAs, and classical ncRNAs, such as ribosomal RNAs, transfer RNAs, and small nucleolar RNAs (Kiss, 2004; Moss et al., 2007; Neguembor et al., 2014). The ncRNAs that are longer than 200 nucleotides are described as long non-coding RNAs (lncRNAs) (Neguembor et al., 2014; Zhao et al., 2015). The transcription of these RNAs plays a critical role in muscle development through the regulation of transcription and post-transcriptional gene expression (Neguembor et al., 2014).

Time-course data for lncRNA expression can help to identify important lncRNAs that regulate muscle development. Catalogs of lncRNAs involved in muscle development have been established for many species. For example, a comparison of lncRNA expression profiles in skeletal muscles of Meishan and long white pigs, at 1, 90, and 180 days of age, found 1,407 differentially expressed lncRNAs (DE-lncRNAs) with consistent patterns of expression between the two breeds, at all three sampling points (Gao et al., 2017). Characterization of lncRNA in developing skeletal muscle of Jianzhou big-eared goats, at 45, 60, and 105 days of gestation, identified 577 lncRNAs that were differentially expressed between these stages (Zhan et al., 2016). Sun et al. (2016) identified 401 DE-lncRNAs between embryonic, neonatal, and adult skeletal muscle, in bovines. They demonstrated that lncMD acts as a competing endogenous RNA to sequester miR-125b, which leads to heightened insulin like growth factor 2 (*IGF2*) expression and thus promotes muscle differentiation (Sun et al., 2016). In chickens, Li et al. (2012) identified the lncRNA profiles in White Leghorn breast muscle at embryonic days 10, 12, 14, and 18 (Li et al., 2012), while Li et al. (2017) profiled the leg muscle transcriptome of the Xinghua chicken at embryonic days 11 and 16 and 1-day post-hatching. They identified 129, 132, and 45 DE-lncRNAs by comparing successive ages within each region (Li et al., 2017).

Muscle development in chicken involves two major stages. Hyperplasia refers to the increase in cell number or muscle fiber number, which mainly occurs in the embryonic period. Hypertrophy refers to the increase in cell size that mainly occurs after birth (Ylihärsilä et al., 2007; Liu et al., 2017; Ouyang et al., 2017). Most previous studies have focused on the expression profile of the lncRNA transcriptome in the embryonic period. Few studies have investigated the whole muscle development, from embryonic to post-hatching periods in the chicken. We systematically investigated the expression profile of the lncRNA transcriptome from embryonic to post-hatching period, using data from our previously published study (Liu et al., 2019) to assemble the transcriptome. We also downloaded data from the Genome Sequence Archive (GSA) at http://bigd.big.ac.cn/gsa (accession no CRA001773.). This work facilitates the systematic exploration of the development-related lncRNA expression signatures in breast muscle and provides new insights into the molecular mechanisms that affect the growth performance of chicken.

## Materials and Methods

### Ethics Statement

All animal experiments were conducted in accordance with the Guidelines for Experimental Animals, established by the Ministry of Science and Technology (Beijing, China). Animal experiments were approved by the Science Research Department of the Shandong Academy of Agricultural Sciences (SAAS) (Ji’nan, China). Ethical approval for animal survival was given by the animal ethics committee of SAAS (No. SAAS-2019-029).

### Animals

The Shouguang chicken eggs were obtained from the experimental farm of the Poultry Institute (PI), Shandong Academy of Agricultural Sciences (SAAS, Ji’nan, China). All eggs were incubated using the normal procedure and chicks were reared in caging, using standard conditions of temperature, humidity, and ventilation, on the farm at the PI, SAAS. Breast muscles were sampled from 12-day embryos (E12), 17-day embryos (E17), 1-day post-hatch chicks (D1), 14-day post-hatch chicks (D14), 56-day post-hatch chicks (D56), and 98-day post-hatch chicks (D98). The sex of each chicken was determined by PCR analysis of the *CHD1* gene (Fridolfsson and Ellegren, 1999). Chickens that gave two PCR products of 600 bp and 450 bp were female, while those with one product of 600 bp were male. We constructed 17 cDNA libraries from breast muscle samples at six developmental stages (E12, E17, D1, D14, D56, and D98). Approximately 5 μg of total RNA was used to deplete ribosomal RNA, in accordance with the Ribo-Zero^TM^ rRNA Removal Kit instructions (Illumina, San Diego, USA). The RNA was then fragmented into small fragments using divalent cations, under high temperature. The fragmented RNAs were reverse-transcribed to create the cDNA, which was used to synthesize U-labeled second-strand DNAs, in a reaction with *E. coli* DNA polymerase I, RNase H, and dUTP. An A-base was then added to the blunt ends of each strand to prepare them for ligation to the indexed adapters. Each adapter contained a T-base overhang to ligate the adapter to the A-tailed fragmented DNA. Single- or dual-index adapters were ligated to the fragments, and size selection was performed using AMPureXP beads. After heat labile UDG enzyme treatment of the U-labeled second-strand DNAs, the ligated products were amplified by PCR using the following conditions: initial denaturation at 95°C for 3 min; eight cycles of denaturation at 98°C for 15 s, annealing at 60°C for 15 s, and extension at 72°C for 30 s; followed by a final extension at 72°C for 5 min. The average insert size for the final cDNA library was 300 bp (± 50 bp). The libraries were sequenced using an Illumina HiSeq 4,000 platform and 300-bp paired-end reads were generated. The datasets were downloaded from the GSA in the Beijing Institute of Genomics (BIG) Data Center, BIG, Chinese Academy of Sciences. These data are publicly accessible at http://bigd.big.ac.cn/gsa (accession no CRA001773).

### Transcript Assembly

Initially, Cutadapt^1^ (Martin, 2011) was used to remove the reads that contained adaptor contamination, low-quality bases, and undetermined bases. Sequence quality was then verified using FastQC v0.10.1 (Beekman et al., 2018)^2^. We used hisat2-2.0.4 (Kim et al., 2015)^3^ to map reads to the genome of Gallus_gallus 5.0 (GCA_000002315.3) (command line: hisat2-1 R1.fastq.gz-2 R2.fastq.gz -S mapped.sam). The mapped reads from each sample were assembled using StringTie-1.3.4 (Pertea et al., 2015)^4^, with default parameters (command line: stringtie -p 2 -G genome.gtf -o output.gtf -l mapped.bam). All transcriptomes obtained from samples were then merged to reconstruct a comprehensive transcriptome using gffcompare^5^. StringTie was then used to assess expression levels for the transcripts by calculating FPKM (fragments per kilobase of transcript per million fragments mapped) (Trapnell et al., 2010) (FPKM = [total_exon_fragments / mapped_reads (millions) × exon_length (kb)]).

### LncRNA Identification

Initially, we discarded transcripts that overlapped with known mRNAs and transcripts that were shorter than 200 bp. We then utilized CPC (Kong et al., 2007) and CNCI (Sun et al., 2013) to predict transcripts with coding potential. All transcripts with a CPC score <−1 and a CNCI score <0 were removed. We also removed any remaining transcripts with similarity to known proteins in the Swiss-Prot database and Pfam database (release 33.1), with an E-value ≤10^–5^. The remaining transcripts with class codes i, j, o, u, or x were considered to be lncRNAs, whereby (i) is a transfrag falling entirely within a reference intron (intronic); (j) is a potentially novel isoform or fragment where at least one splice junction is shared with a reference transcript; (o) shows generic exonic overlap with a reference transcript; (u) is an unknown, intergenic transcript (intergenic); and (x) shows exonic overlap with a reference transcript on the opposite strand (antisense).

### Differential Expression Analysis of lncRNAs and mRNAs

StringTie (Pertea et al., 2015) was used to determine expression levels of lncRNAs and mRNAs by calculating FPKM. The differentially expressed lncRNAs (DE-lncRNAs) and mRNAs (DE-mRNAs) were selected with a log2 (fold change) ≥1 or log2 (fold change) ≤−1 and with statistical significance (*q* value ≤ 0.05), using R package Ballgown (Frazee et al., 2015).

### Time Series Expression Profile Clustering

Co-expressed lncRNAs and mRNAs were clustered using STEM (Short Time-Series Expression Miner, version 1.3.11) (Ernst and Bar-Joseph, 2006). Expression profiles of lncRNA transcripts and mRNAs were clustered based on log2 (FPKM values) and their correlation coefficients. The maximum unit change in model profiles between time points was adjusted to 2 and the maximum number of model profiles was adjusted to 30. The statistical significance of the actual number of lncRNA transcripts and mRNAs in each profile versus the expected number was computed using the algorithm proposed by Ernst and Bar-Joseph (2006).

### Target Gene Prediction and Functional Enrichment Analysis

The lncRNAs and mRNAs in the same profile, with the same expression pattern, were selected for prediction of the potential *cis* and *trans* interactions. We used the UCSC Genome Bioinformatics tool to identify mRNAs located approximately 100 kb upstream and downstream of lncRNAs and to determine the potential that the lncRNA was *cis* acting. To classify lncRNA *trans* target genes, the RIsearch software (Wenzel et al., 2012) was used to assess the impact of lncRNA binding on complete mRNA molecules (Pearson Correlation Coefficient >0.8). Based on the regulatory relationship between lncRNA and mRNA, genes of known function can be used to predict the function of unknown lncRNAs. The genes regulated by lncRNAs were used to form a gene list that was input into DAVID software (Sherman and Lempicki, 2009) for GO analysis. A KEGG enrichment analysis of the predicted target genes was performed with KOBAS software (Xie et al., 2011) using a hypergeometric test. The GO terms and KEGG pathways with *P* < 0.05 were considered to be significantly enriched.

### Quantitative Reverse Transcription PCR Confirmation

To confirm our differential expression results, we conducted quantitative reverse transcription PCR (qRT-PCR) for six randomly selected lncRNAs (*MSTRG.1790.1*, *MSTRG.1967.1*, *MSTRG.11775.1*, *MSTRG.12254.1*, *MSTRG.13530.1*, and *MSTRG.34078.1*). Total RNA was extracted using TRIzol reagent (Invitrogen, Carlsbad, CA, United States). Add 1 ml of TRIzol^TM^ Reagent per 50–100 mg of muscle tissue to the sample and homogenize using a homogenizer. Incubate for 5 min to permit complete dissociation of the nucleoproteins complex. Add 0.2 ml of chloroform per 1 ml of TRIzol^TM^ Reagent used for lysis and then securely cap the tube. Incubate for 2–3 min. Centrifuge the sample for 15 min at 12,000 × *g* at 4°C. Transfer the aqueous phase containing the RNA to a new tube and pipette the solution out. Add 0.5 ml of isopropanol to the aqueous phase, per 1 ml of TRIzol^TM^ Reagent used for lysis. Incubate for 10 min. Centrifuge for 10 min at 12,000 × *g* at 4°C. Total RNA precipitate forms a white gel-like pellet at the bottom of the tube. Discard the supernatant and wash the RNA with 75% ethanol. Finally, resuspend the pellet in 20–50 μl of RNase-free water. The total RNA quantity and purity were analyzed using Bioanalyzer 2100 and RNA 1000 Nano LabChip Kit (Agilent, CA, United States) with RIN number >7.0. First-strand cDNA was synthesized from 1 μg of total RNA, random primers, and oligo(dT) primers using the Transcriptor First Strand cDNA synthesis Kit (TAKARA, Dalian, China) following the manufacturer instructions. Any residual genomic DNA was removed by a treatment with DNaseI (Invitrogen, Burlington, ON, Canada) prior to cDNA synthesis. The cDNA was subsequently used for qRT-PCR analyses on an ABI 7500 Detection System (Applied Biosystems, Foster City, CA, United States). The primers were designed using Primer Premier version 5.0 (PREMIER Biosoft, Palo Alto, CA, United States), as listed in Supplementary Table 1. The amplification was performed in triplicate in a total volume of 20 μl, containing 10 μl of 2 × KAPA SYBR FAST qPCR Master Mix (KAPA Biosystems, Boston, MA, United States), 1 μl of the diluted cDNA, and 0.5 μl of each primer, and 0.4 μl of ROX Low and 7.6 μl of PCR-grade water. The real-time PCR program started with denaturing at 95°C for 3 min, followed by 40 cycles of 95°C for 3 s and 60°C for 34 s. The qRT-PCR was performed following the instructions for the ABI 7500 with default parameters. The 2^–Δ^ ^Δ^ ^*Ct*^ method (Livak and Schmittgen, 2001) was used to calculate the relative lncRNA abundance. The beta actin gene (*ACTB*) was used as the housekeeping gene. Three independent replicates were used for each assay and data were presented as means ± SD.

## Results

### Identification of lncRNAs in Chicken Breast Muscle

**FIGURE 1:**
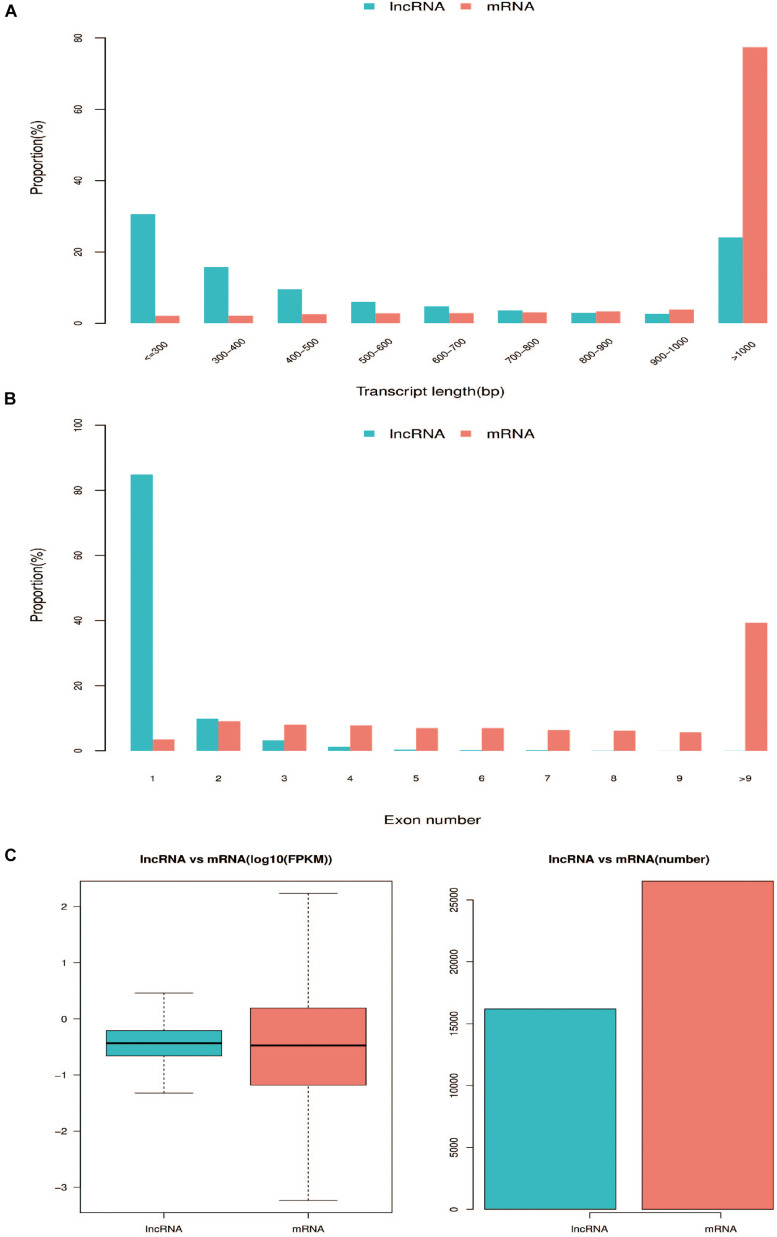
Comparison of genomic features between predicted lncRNAs and mRNAs. **(A)** Distribution of transcript lengths in lncRNAs and mRNAs. **(B)** Distribution of the number of exons in lncRNAs and mRNAs. **(C)** Expression level of lncRNAs and mRNAs.

A total of 1,424,615,174 raw reads were generated in the 17 libraries and 1,375,301,128 clean reads were obtained after adaptor sequences and low quality reads were discarded (Supplementary Table 2) (Liu et al., 2019). We mapped the clean reads to the chicken reference genome, Gallus_gallus 5.0, and then developed a highly stringent filtering pipeline to discard transcripts that did not have all the characteristics of lncRNAs. Our pipeline yielded 14,793 lncRNA transcripts (Supplementary Table 3). The length of these transcripts ranged from 196 to 58,084 bp, with 29.78, 47.11, and 23.11% of lncRNA transcripts having a length of < 300 bp, 300–1000 bp, and > 1000 bp, respectively (Figure 1A). The lncRNAs had fewer exons than mRNAs (Figure 1B) and were expressed at lower levels than mRNAs (Figure 1C). We identified 2,858 lncRNA transcripts and 4,282 mRNAs that were differentially expressed during muscle development [*q* ≤ 0.05, log2 (fold change) ≥ 1 or log2 (fold change) ≤ −1] (Supplementary Table 4).

### STEM Analysis and Target Prediction of Differentially Expressed lncRNA and mRNA Profiles

**FIGURE 2:**
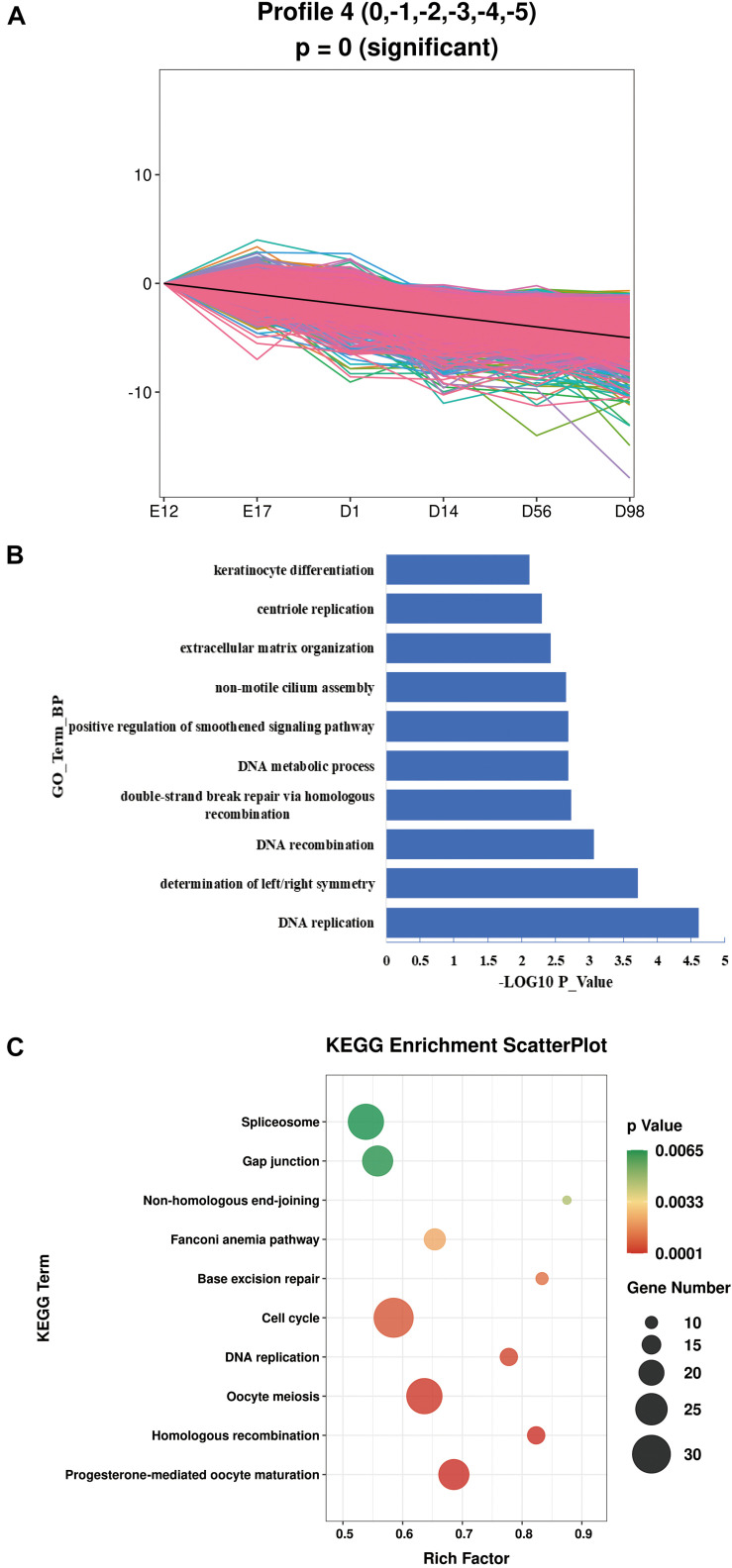
The GO enrichment and KEGG pathway analysis of profile 4. **(A)** Details of profile 4. **(B)** The top 10 biological processes in profile 4. **(C)** The top 10 KEGG pathways in profile 4.

**FIGURE 3:**
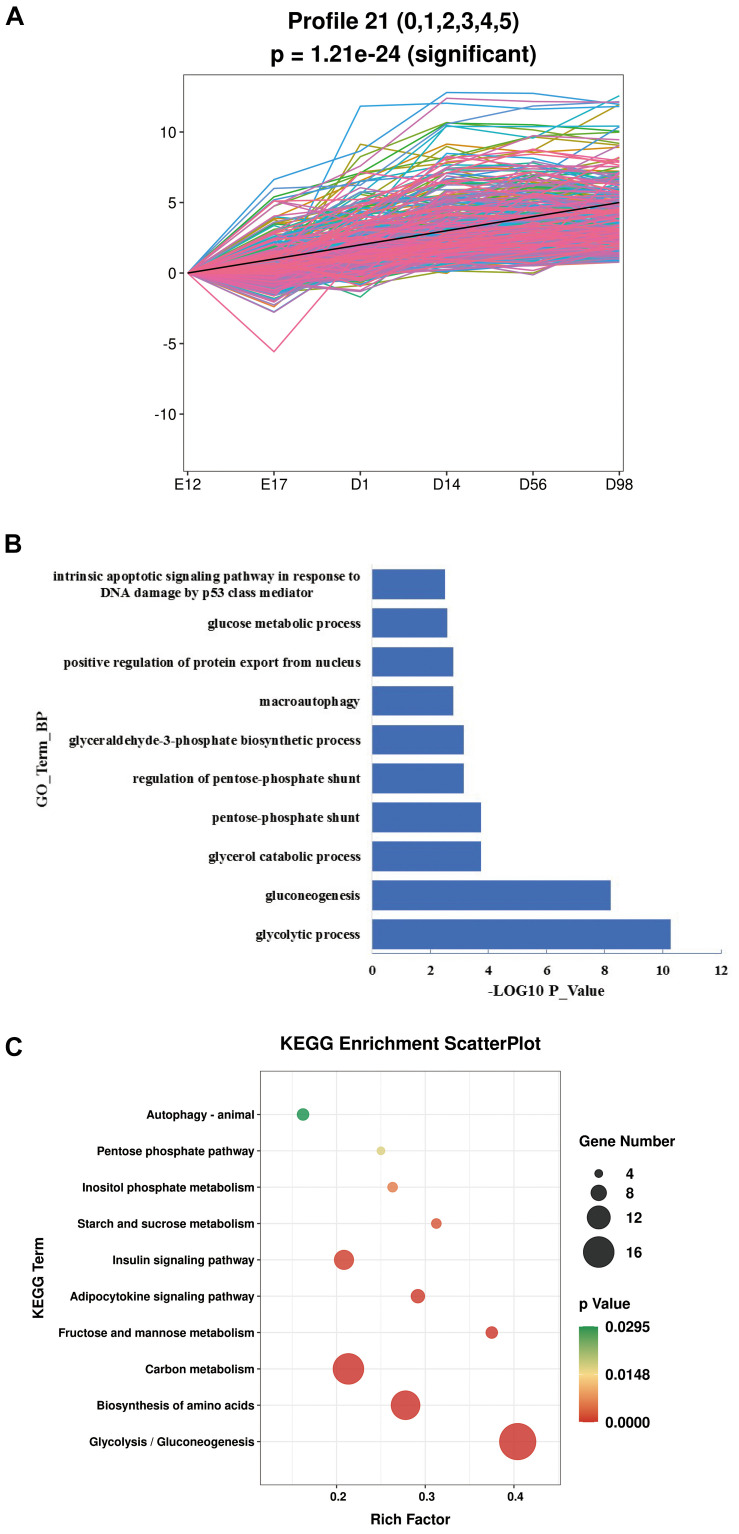
The GO enrichment and KEGG pathway analysis of profile 21. **(A)** Details of profile 21. **(B)** The top 10 biological processes in profile 21. **(C)** The top 10 KEGG pathways in profile 21.

As our data were collected at different time points, STEM was used to cluster and visualize possible changes in the profiles of 2,858 lncRNAs and 4,282 mRNAs at six time points (Supplementary Figure 1). This also helped to identify the co-expressed lncRNAs and mRNAs. We identified two profiles with opposite expression patterns. Profile 4 had a downregulated pattern and contained 1,383 lncRNAs and 1,851 mRNAs (Figure 2A and Supplementary Table 5). Profile 21 had an upregulated pattern and contained 39 lncRNAs and 362 mRNAs (Figure 3A and Supplementary Table 6). Since the co-expressed lncRNAs and mRNAs may interact, we predicted the potential *cis* and *trans* interactions between lncRNAs and mRNAs in the same profile, based on the STEM analysis. The results showed that 1785 mRNAs were regulated by 1,353 lncRNAs through *trans* interaction in profile 4 (Pearson Correlation Coefficient >0.8) (Supplementary Table 7). Among these lncRNAs, *MSTRG.30304.1*, *MSTRG.31306.1*, *MSTRG.30819.1*, *MSTRG.39432.1*, and *MSTRG.35315.1* were highly expressed and some of the genes regulated by these lncRNAs contribute to muscle development (Table 1). For example, *EPHB1* is a key factor in skeletal muscle satellite cell activation and *PPP1R12B* contributes to the regulation of actin cytoskeleton organization. In profile 21, 233 mRNAs were regulated by 35 lncRNAs through *trans* interaction (Pearson Correlation Coefficient >0.8) (Supplementary Table 8) and *MSTRG.31694.1*, *MSTRG.30597.1*, *MSTRG.32189.1*, *MSTRG.30605.1*, and *MSTRG.36552.1* showed abundant expression. *Trans* interaction prediction showed that *AQP3*, *GIT1*, and *PGM2L1* were extensively regulated by these lncRNAs (Table 2).

**TABLE 1:** The top five differentially expressed lncRNAs and their target genes in profile 4.

lncRNA_name	E12(FPKM)	E17(FPKM)	D1(FPKM)	D14(FPKM)	D56(FPKM)	D98(FPKM)	Top 5 target genes
MSTRG.30304.1	100.37	52.23	58.76	24.16	17.52	33.26	*AIDA, ARHGEF17, CRAC1A, EDNRA, NUDT21*
MSTRG.31306.1	86.24	61.68	54.63	21.72	7.57	20.92	*ENSGALG00000035784, EXT1, PRKAR1B, PHLPP1, STK32C*
MSTRG.30819.1	73.78	62.93	100.11	1.79	13.66	5.90	*GLIS2, GFRA4, MYCT1, MRPS23, SH3PXD2A*
MSTRG.39432.1	68.46	92.75	6.10	0.70	0.32	0.55	*EPHB1, EYA1, PKP2, TRIM36, MYO10*
MSTRG.35315.1	32.83	9.06	14.68	0.94	1.35	1.07	*NCL, PIGS, PCDHGC3, PPP1R12B, POLA1*

**TABLE 2:** The top five differentially expressed lncRNAs and their target genes in profile 21.

lncRNA_name	E12(FPKM)	E17(FPKM)	D1(FPKM)	D14(FPKM)	D56(FPKM)	D98(FPKM)	Top 5 target genes
MSTRG.31694.1	27.95	37.99	31.10	64.58	99.32	159.98	*AQP3, GIT1, PGM2L1, VWF, SLC25A39*
MSTRG.30597.1	2.83	3.43	23.79	28.11	40.36	66.44	*AQP3, BACH2, GSK3B, MAPRE3, FLII*
MSTRG.32189.1	1.91	2.38	4.16	6.56	12.19	16.56	*AQP3, BFIV21, SLC25A39, PPM1J, PRKAA2*
MSTRG.30605.1	0.42	0.33	2.37	4.65	7.14	13.11	*AQP3, GIT1, PGM2L1, PPM1J, VWF*
MSTRG.36552.1	0.53	1.21	5.23	9.59	7.34	12.32	*ADIPOR2, ATG2B, ENSGALG00000005350, FBXL21, IDE*

### Functional Enrichment Analysis

In order to explore the function of the lncRNAs identified in this study, we performed GO and KEGG pathway analyses for the lncRNA targets. For profile 4, with the downregulated pattern, lncRNAs mainly regulated genes associated with the cell proliferation process (Supplementary Table 9). The top 10 biological processes in profile 4 were enriched in DNA replication, DNA recombination, DNA metabolic process, and centriole replication (Figure 2B). The top 10 pathways enriched in profile 4 were mainly involved in cell cycle, DNA replication, homologous recombination, spliceosome, and base excision repair (Figure 2C). For profile 21, with the upregulated pattern, lncRNAs mainly regulated genes associated with metabolism (Supplementary Table 10). The GO terms involved in metabolism processes were significantly enriched and included glycolytic process, gluconeogenesis, glycerol catabolic process, and positive regulation of protein export from the nucleus (Figure 3B). Pathways such as glycolysis/gluconeogenesis, biosynthesis of amino acids, pentose phosphate pathway, and insulin signaling were also significantly enriched (Figure 3C).

### Validation of Differentially Expressed Lncrnas by Quantitative Reverse Transcription PCR

**FIGURE 4:**
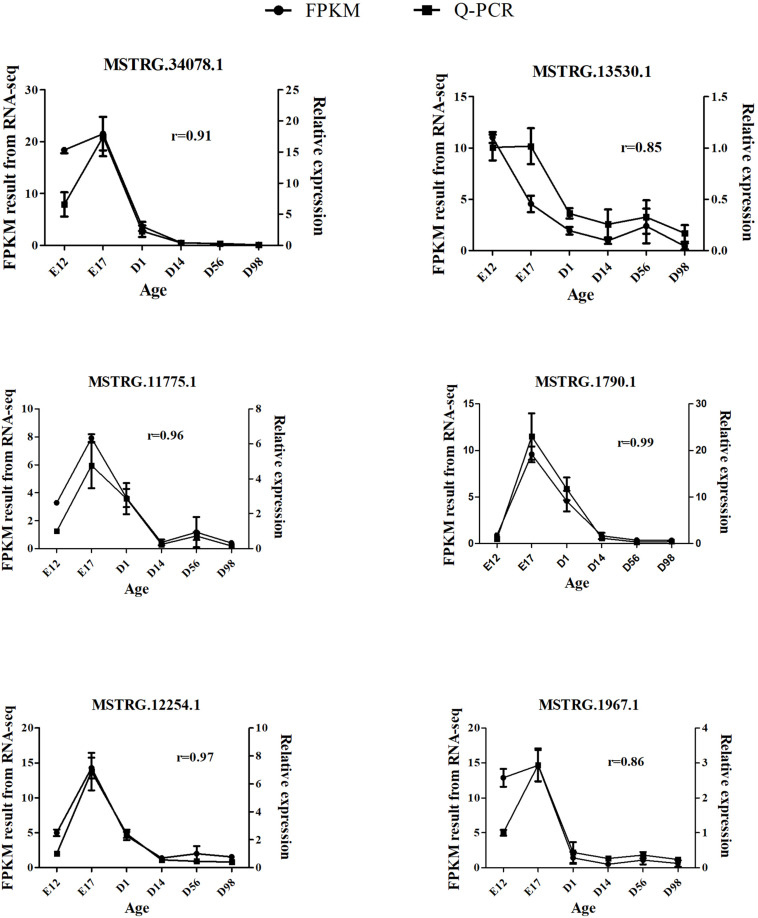
Validation of six DE-lncRNAs by qRT-PCR. The *r* value represents the Pearson correlation coefficient between two methods.

The qRT-PCR assays were conducted to validate DE-lncRNAs from the RNA-seq data. We randomly selected six DE-lncRNAs and examined their expression patterns at six developmental stages. The results confirmed that the six lncRNAs were expressed during all six developmental stages (Figure 4) and showed differential expression at different stages. In addition, the qRT-PCR confirmed that the expression patterns of the six lncRNAs were consistent with their expression levels determined using the RNA-seq data. These results indicate that our pipeline is accurate in the identification of putative lncRNAs.

## Discussion

The muscle development process is the result of precise regulation of genes, ncRNAs, and other factors (Nakasa et al., 2010; Moncaut et al., 2013; Huang et al., 2018). lncRNA is an important member of the group of ncRNAs and plays a critical role in muscle development through the regulation of transcriptional and post-transcriptional gene expression. Previous studies have identified numerous lncRNAs in livestock (such as pig, cattle, and chicken) and have demonstrated that they play key roles in specific developmental stages for different tissues (such as liver, muscle, and adipose tissue) (Muret et al., 2017; Kern et al., 2018; Kosinska-Selbi et al., 2020). In recent years, several studies have been performed on lncRNA identification and pathway regulation in chicken skeletal muscle development (Li et al., 2012, 2017; Ren et al., 2018). The mechanisms by which some lncRNAs regulate muscle development have also been revealed (Cai et al., 2017; Ren et al., 2017). However, these studies have only focused on the embryonic or post-hatching period. There is still a lack of understanding of the molecular mechanisms that underlie skeletal muscle development throughout this period. In this study, we identified the lncRNA profiles in six representative developmental stages of breast muscle in Shouguang chicken. Since lncRNAs are generally expressed at low levels, which can be hard to separate from background noise, the use of 17 biological replicates helped to verify the reproducibility of the results. Any three biological replicates with FPKM values of greater than zero were considered to represent reliable lncRNA expression. Based on the study criteria, a total of 14,793 lncRNA transcripts were identified in the breast muscle samples. A total of 2858 were differentially expressed during muscle development [*q* < 0.05, log2 (fold change) ≥1 or log2 (fold change) ≤−1]. When compared with mRNAs, the lncRNAs were shorter, had fewer exons, and lower expression, which is consistent with previous reports (Zhan et al., 2016; Sulayman et al., 2019). These data provide a valuable resource for the analysis of molecular mechanisms that underlie the development of chicken skeletal muscle and post-transcriptional regulation.

As a rule, the guilt-by-association principle is applied. This states that genes that share the same function or are involved in the same regulatory pathway will tend to present similar expression profiles and hence form clusters or modules in the network. Thus, within the same module, genes of known function can be used to predict the function of co-expressed, unknown genes (Serin et al., 2016). Transcriptional regulation by lncRNAs may work in either *cis* or *trans* and may negatively or positively control mRNA expression. Therefore, we performed a STEM analysis to identify transcripts (lncRNAs and mRNAs) with the same expression profile and used them for further *cis* and *trans* regulatory prediction. The analytical method applied in our study may be more inclined to focus on lncRNAs that show positive regulation of mRNA expression. For profile 4, with the downregulated pattern, 1785 mRNAs were regulated by 1353 lncRNAs through *trans* regulation. The functional analysis showed that lncRNAs mainly regulated genes associated with the cell proliferation process, such as cell cycle, DNA replication, and homologous recombination. This is similar to previous research on goat muscle development, from gestation to birth, which showed that genes with downregulated patterns were also involved in the cell proliferation process (Zhan et al., 2018). As shown in Table 1, the abundantly expressed lncRNAs can regulate protein coding genes that are involved in cell proliferation, as discussed below. The *EDNRA* (endothelin receptor A) gene, which causes cellular proliferation, was regulated by *MSTRG.30304.1*. The expression of *EDNRA* has been shown to be positively correlated with muscle proliferation (Darrah et al., 2010). The *PPP1R12B* (Protein phosphatase 1 regulatory subunit 12B) gene was regulated by *MSTRG.35315.1* and has a potential role in muscle cell proliferation (Montén et al., 2015), as observed in a study that showed higher levels of expression in high muscle growth bulls than in low muscle growth bulls (Sudre et al., 2005). As the target of *MSTRG.39432.1*, the *EPHB1* transmembrane receptor has been shown to be expressed early on in myogenic development (Alonso-Martin et al., 2016). The Myo10 protein can act as a link between integrins and microtubules. Its upregulation may be a response to a mechanism in mdx skeletal muscle that reinforces the interactions between the myofiber cytoskeleton and the plasma membrane (Marotta et al., 2009). The Circ-calm4 protein can promote smooth muscle proliferation by the regulation of *Myo10* (Zhang et al., 2020).

The classical pathways involved in muscle development were also identified in profile 4. The Wnt signaling pathway plays an essential role in embryonic muscle development and in the maintenance of skeletal muscle homeostasis in the adult. During embryonic development, Wnt signals control the expression of myogenic regulatory factors (MRFs), which are essential for myogenic lineage progression (von Maltzahn et al., 2012). The Wnt proteins typically bind to Frizzled receptors (Fzd), and when canonical Wnts bind to their respective receptors, heterotrimeric G proteins and Dsh become activated. This leads to the recruitment of axin to the Fzd coreceptor low-density lipoprotein receptor-related protein (LRP). Subsequently, the degradation complex is inactivated and β-catenin accumulates in the cytoplasm. On its release, β-catenin translocates into the nucleus and binds members of the TCF and LEF family of transcription factors. The β-catenin protein functions as a transcriptional coactivator to induce context-dependent Wnt/β-catenin target genes, whose transcription controls several biological processes, such as early myogenesis in the somite (Sethi and Vidal-Puig, 2010; von Maltzahn et al., 2012). We identified *WNT4*, *LRP6*, *TCF7L2*, *Lef-1*, and *FZD3* (Table S9), which are involved in the Wnt signaling pathway, in profile 4. The *Wnt4* gene is expressed in the dorsal regions of the neural tube and induces somitic myogenesis in cooperation with Shh signaling from the notochord (Münsterberg et al., 1995). The Wnt4 protein has been shown to increase the number of fast MyHC fibers in the chick limb bud (Takata et al., 2007). It has also been found that Wnt signals, which directly affect the Lef1 transcriptional activator and Pitx2 transcription factor activity, determine the number of premyogenic Pax3/Pax7 cells (Abu-Elmagd et al., 2010). Muendlein et al. (2011) hypothesized that TCF7L2 may directly influence the regulation of smooth muscle cell proliferation and the NF_KB pathway through the Wnt signaling pathway. Srivastava et al. (2015) demonstrated that loss of LRP6 activity results in loss of vascular smooth muscle cell (VSMC) differentiation via reduced TCF7L2-dependent inhibition of Sp1. The TGF-β pathway plays an important role in cell proliferation, differentiation, morphogenesis, and tissue homeostasis and regeneration, in addition to being involved in a number of severe diseases (Massagué, 2012). In skeletal muscle, TGF-β superfamily members have been shown to have potent effects on both muscle development and postnatal skeletal muscle mass. Gene expression in muscle cells is reprogrammed by TGF-β, which results in an alteration in proliferative control and a potent inhibition of the program of gene expression that underlies myogenic differentiation. For example, as key orchestrators of muscle gene expression, the MRFs have been shown to be targeted by TGF-β signaling (Kollias and McDermott, 2008). The TGF-β superfamily consists of over 50 structurally related ligands, many of which fall into three major subfamilies: TGF-β, bone morphogenic protein (BMP), and activin (Feng and Derynck, 2005). For instance, TGFβ1 has been shown to have an inhibitory effect on myoblast differentiation (Cusella-De Angelis et al., 1994), while BMP signaling is a positive regulator of muscle mass (Sartori et al., 2013). *BAMBI* gene encodes a transmembrane protein and is involved in multiple physiological and pathological processes, along with BMP. The shuttling of BAMBI between the cytosol and membrane is required for skeletal muscle development and regeneration (Yao et al., 2019). Myostatin (MSTN) inhibits cellular differentiation of developing somites during the embryonic stage and diminishes myofibrillar growth during the post-embryonic period, through ACVR2A (Activin receptor type IIA) and ACVR2B (Activin receptor type IIB) (Bhattacharya et al., 2019). In this study, we identified *BMP5*, *BAMBI*, *ACVR2A*, and *ACVR2B* (Table S9), which are involved in the TGF-β signaling pathway. Moreover, several studies have suggested that TGFβ, downstream of canonical Wnt signaling, may be required to instruct cell cycle arrest in proliferating muscle cells as a prerequisite for terminal differentiation (Girardi and Le Grand, 2018). A previous study has reported that the total number of skeletal myofibers is defined by hyperplasia during embryogenesis (Sporer et al., 2011). Our results also showed that downregulated lncRNAs may contribute to hyperplasia through cell proliferation at the lncRNA level.

For profile 21, with an upregulated pattern, 233 mRNAs were regulated by 35 lncRNAs through *trans* regulation. The functional annotation showed that the lncRNA targets mainly took part in metabolism. The following pathways and GO terms were significantly enriched in profile 21: glycolysis/gluconeogenesis, biosynthesis of amino acids, insulin signaling pathway, and glycerol catabolic process. As shown in Table 2, the lncRNAs were involved in metabolism by the regulation of protein coding genes. The GSK3B protein (glycogen synthase kinase 3 beta) is a growth signaling-sensitive kinase and can regulate mitochondrial energy metabolism by affecting the stability and activity of PGC-1a. A previous study showed that *AKT1* mediates muscle growth and protein upregulation signals by regulating *GSK3B* (Cassano et al., 2009). This is consistent with our study that showed that *MSTRG.30597.1* may contribute to muscle development by the regulation of *GSK3B*. The AMP-activated protein kinase (AMPK) is a metabolic master switch that regulates glucose and lipid metabolism (Dervishi et al., 2011). The *PRKAA2* gene, which can be regulated by *MSTRG.32189.1*, encodes the α2 catalytic subunit of AMPK. Single SNP (single nucleotide polymorphism) and haplotype analyses have revealed associations between the *PRKAA2* genotypes and loin muscle area (Lin et al., 2010). The *GIT1* gene is a member of the *GIT* family, which plays a key role in the MAPK pathway (Zhao et al., 2020). This pathway has been demonstrated to be a major regulator of skeletal muscle development (Keren et al., 2006). In this study, the *GIT1* gene has been identified as the potential target for regulation by *MSTRG.31694.1* and *MSTRG.30605.1*. Aquaporin 3 (AQP3) belongs to a family of transmembrane channels that are important in transporting water, glycerol, and other small molecules across the cell membrane. The AQP3 protein can be regulated by *MSTRG.32189.1*, *MSTRG.31694.1*, *MSTRG.30597.1*, and *MSTRG.30605.1*. Glycerol is transported by AQP3 and is necessary for cell energy generation and lipid synthesis, which belong to cell biological processes (Li et al., 2016). The *AQP3* gene has been reported to be co-expressed with AQP4 in various kinds of muscle, including skeletal muscle (Wang et al., 2003). Therefore, AQP3 may play a key role in muscle development through the regulation of skeletal muscle contraction or metabolism. We have indicated that the lncRNAs with an upregulated pattern may affect muscle development after hatching by regulation of metabolism. This is consistent with a previous study that showed that muscle mass increases by hypertrophy (increased cellular protein content) after hatching and is controlled by synthesis of muscle proteins or their degradation (Braun and Gautel, 2011).

## Conclusion

In summary, we have described the lncRNA and mRNA profiles of chicken breast muscle at time points E12, E17, D1, D14, D56, and D98 and identified 2858 DE-lncRNAs and 4282 mRNAs. We performed co-expression analysis for the lncRNAs and mRNAs, using STEM, and then predicted the *cis* and *trans* regulatory interactions between the lncRNAs and mRNAs in the same profile. The results showed that only *trans* regulation reached significant levels. Functional analysis of the mRNAs regulated by lncRNAs showed that lncRNAs in profile 4, with a downregulated pattern, contributed to the cell proliferation process. The lncRNAs in profile 21, with an upregulated pattern, were mainly involved in metabolism. Our study provides new insights into the discovery and annotation of lncRNAs associated with breast muscle development in chickens. Further research is required to validate the functions of these lncRNAs and their targets in chicken skeletal muscle development at the cellular level.

## Data Availability Statement

Publicly available datasets were analyzed in this study. This data can be found here: The datasets were obtained from our previously published study (Liu et al., 2019) and downloaded from the GSA in BIG Data Center, Beijing Institute of Genomics (BIG), Chinese Academy of Sciences, and are publicly accessible at http://bigd.big.ac.cn/gsa (accession no CRA001773.).

## Ethics Statement

The animal study was reviewed and approved by Animal Ethics Committee of SAAS.

## Author Contributions

JL, YZ, and XH performed the experiments, data analysis, and manuscript writing. HH, QL, WL, and JY contributed to the animal experiments and data analysis. FL and DC designed the experiments, and supervised and coordinated the study. All authors reviewed the manuscript.

## Conflict of Interest

The authors declare that the research was conducted in the absence of any commercial or financial relationships that could be construed as a potential conflict of interest.

## Footnotes

1 http://cutadapt.Readthedocs.io/en/stable/

2 http://www.bioinformatics.babraham.ac.uk/projects/fastqc/

3 https://daehwankimlab.github.io/hisat2/

4 http://ccb.jhu.edu/software/stringtie/

5 https://github.com/gpertea/gffcompare

## Supplementary Material

Supplementary Figure 1 STEM analysis of transcript profiles. STEM uses a method of analysis that takes advantage of the number of genes being large and the number of time points being small to identify statistically significant temporal expression profiles and the genes associated with these profiles. The black discount in the box indicates the overall trend of the expression of all genes in the profile. The number in the top left-hand corner of a profile box is the profile ID number. The colored boxes represent significantly enriched profiles (*p* value < 0.05). The boxes are sorted from smallest to largest by *P* value.

Supplementary Table 1 Primers used in this study (XLSX).

Supplementary Table 2 Overview of raw data output and quality assessment. (XLSX).

Supplementary Table 3 The lncRNA transcripts identified in this study. (XLSX).

Supplementary Table 4 List of identified differentially expressed transcripts from pairwise comparisons. (XLSX).

Supplementary Table 5 The transcripts in profile 4. (XLSX).

Supplementary Table 6 The transcripts in profile 21. (XLSX).

Supplementary Table 7 Potential *trans-*target genes of DE-lncRNAs in profile 4 (XLSX).

Supplementary Table 8 Potential *trans-*target genes of DE-lncRNAs in profile 21 (XLSX).

Supplementary Table 9 Functional enrichment analysis of genes regulated by lncRNAs in profile 4 (XLSX).

Supplementary Table 10 Functional enrichment analysis of genes regulated by lncRNA in profile 21 (XLSX).
