# Supplementary figures and images for: Transcriptome Analysis Reveals the Profile of Long Non-coding RNAs During Chicken Muscle Development
Source: Front Physiol. 2021 May 10;12:660370. doi: 10.3389/fphys.2021.660370 (PMC8141850; doi:10.3389/fphys.2021.660370)

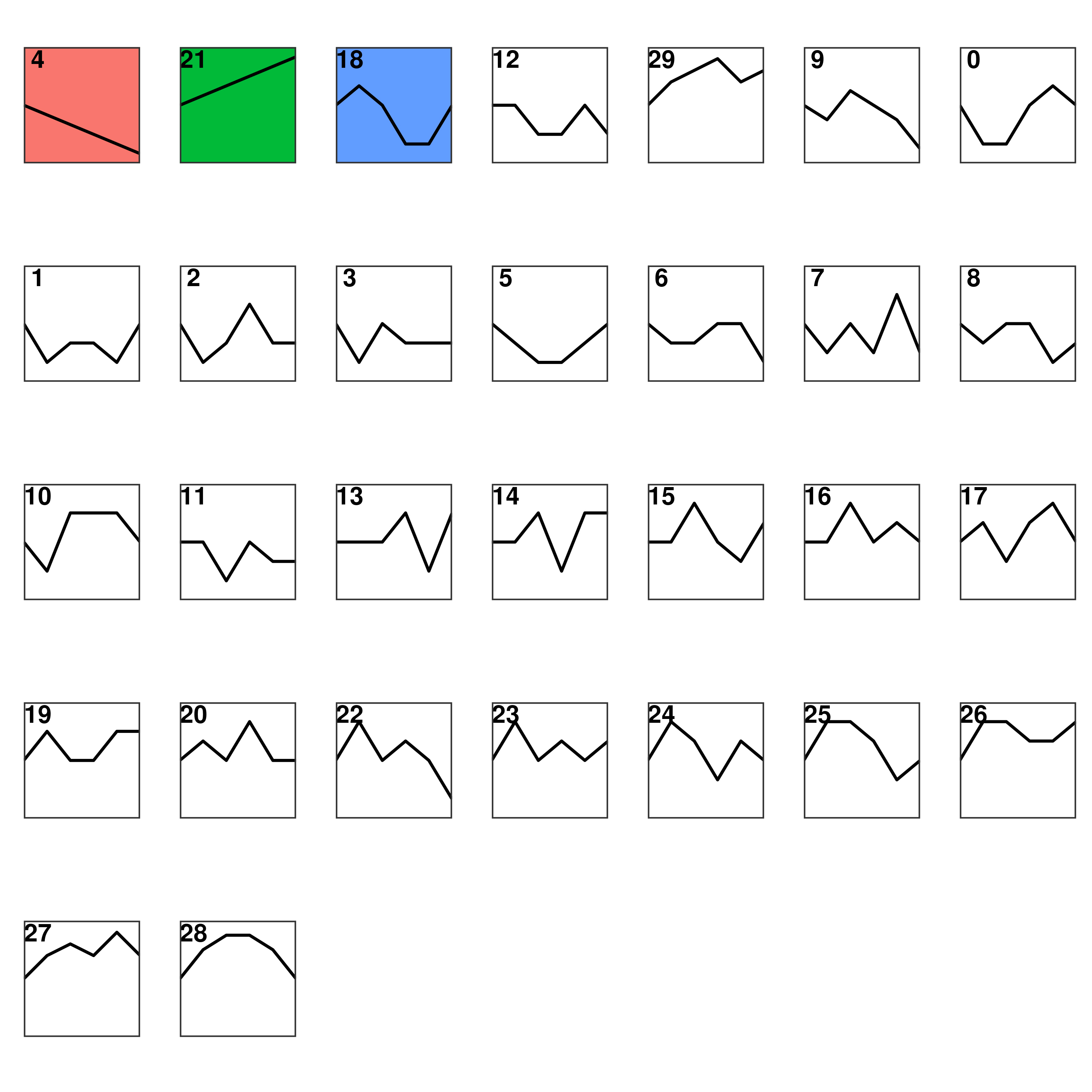

Supplement: Supplementary Figure 1 — STEM analysis of transcript profiles. STEM uses a method of analysis that takes advantage of the number of genes being large and the number of time points being small to identify statistically significant temporal expression profiles and the genes associated with these profiles. The black discount in the box indicates the overall trend of the expression of all genes in the profile. The number in the top left-hand corner of a profile box is the profile ID number. The colored boxes represent significantly enriched profiles (p value < 0.05). The boxes are sorted from smallest to largest by P value. [file Image_1.JPEG]
